# Long-term outcomes of SLIC 2 ligamentoplasty for chronic static scapholunate dissociation: a minimum 5-year follow-up study

**DOI:** 10.1016/j.jham.2026.100493

**Published:** 2026-08-17

**Authors:** Meheddin Rémy Dubian, Jean-Baptiste de Villeneuve Bargemon, Ardouin Ludovic, Philippe Bellemère, Marc Leroy

**Affiliations:** a Department of Hand Surgery, Plastic and Reconstructive Surgery of the Limbs - La Timone University Hospital - Assistance Publique Hôpitaux de Marseille, Marseille, France; b Department of Orthopaedic and Trauma Surgery, AP–HM, Hôpital de la Timone, Marseille, France; c Institute of Movement and Locomotor System (IML), Sainte-Marguerite Hospital (University), 270 Boulevard Sainte-Marguerite, Marseille, France; d Wrist and Hand Academy for Research in the Mediterranean (WHARM), France; e Institut de la Main Nantes-Atlantique, Santé Atlantique, avenue Claude Bernard, Saint-Herblain, 44800, France

**Keywords:** Scapholunate dissociation, Ligamentoplasty, SLIC 2, Wrist instability, SLAC wrist, Long-term follow-up

## Abstract

**Introduction:**

Chronic static scapholunate dissociation remains a therapeutic challenge, and the long-term ability of ligamentoplasty procedures to maintain carpal correction is uncertain. Our hypothesis was that SLIC 2 ligamentoplasty may provide sustained clinical improvement despite partial loss of radiological correction over time. We report the minimum 5-year outcomes of this technique in patients with reducible chronic static scapholunate dissociation.

**Methods:**

This retrospective case series included seven patients who underwent SLIC 2 ligamentoplasty between April 2014 and June 2019 for Garcia-Elias stage 4 reducible chronic static scapholunate dissociation. All patients were reassessed after a minimum follow-up of 5 years. Preoperative and 1-year clinical and radiological data were collected from medical records, and final outcomes were assessed during standardized long-term reassessment. Radiological assessment included the scapholunate gap, scapholunate angle, capitolunate angle, posterior scaphoid subluxation, and posterior radioscaphoid angle. Clinical assessment included pain, grip strength, range of motion, QuickDASH, PRWE, and Mayo Wrist Score.

**Results:**

At a median follow-up of 72 months (IQR, 69.5–78), the initial radiological correction was progressively lost. Compared with immediate postoperative values, the median scapholunate gap increased from 2.0 mm (IQR, 1.8–2.1) to 4.2 mm (IQR, 4.0–4.4) (p = 0.016), and the scapholunate angle increased from 50° (IQR, 49–64.5) to 80° (IQR, 76–86) (p = 0.016). Despite this radiological recurrence, clinical outcomes remained improved. Pain during effort decreased from 5.5 (IQR, 3.8–7.2) to 2 (IQR, 1–3) (p = 0.031), PRWE improved from 49.5 (IQR, 35.8–52.3) to 16 (IQR, 11.3–23.8) (p = 0.031), and Mayo Wrist Score improved from 70 (IQR, 67.5–77.5) to 80 (IQR, 72.5–82.5) (p = 0.016). One patient developed progressive SLAC arthritis.

**Conclusion:**

SLIC 2 ligamentoplasty provided sustained clinical improvement at more than 5 years despite progressive loss of the initial radiological correction. These findings should be interpreted cautiously given the small and highly selected cohort, but suggest that SLIC 2 may remain an option in selected patients with reducible chronic static scapholunate dissociation.

**Level of evidence:**

IV

## 1 Introduction

Scapholunate instability encompasses a broad spectrum of lesions, with multiple patterns and severities of instability 1, 2, 3, 4. Accordingly, treatment options vary according to the stage and extent of the lesion, ranging from arthroscopic capsuloligamentous repair^5^ to more comprehensive ligament reconstruction procedures.^6^^,^^7^ Garcia-Elias and colleagues described the potential progression of scapholunate lesions from predynamic instability to the most advanced stages.^8^ Arthroscopic dorsal capsuloligamentous repair is insufficient to treat static instability. This seems to be confirmed by a recent meta-analysis showing superiority of tenodesis in terms of radiographic results.^9^ Although capsuloligamentous repair may remain feasible in acute and less severe lesions, ligamentoplasty appears to be indicated in chronic static instability.^5^^,^^7^^,^^10^

Several ligamentoplasty procedures have been described; however, a recent scoping review highlighted the substantial heterogeneity of studies on chronic high-grade scapholunate instability and found no evidence supporting the superiority of any single surgical technique.^11^ Ligamentoplasty techniques are generally considered to provide better correction and reduction of scapholunate deformity, but this benefit is often obtained at the cost of significant postoperative stiffness.^7^^,^^12^^,^^13^ Several ligamentoplasty procedures have been described. The SLIC technique reconstructs the dorsal intercarpal ligamentous complex and has shown encouraging preliminary results in both dynamic and static stages, in line with expected functional outcomes.^12^^,^^14^^,^^15^ However, because the available follow-up was limited to three years, the long-term maintenance of reduction and correction of scapholunate instability remains uncertain.

The primary objective of the study was to determine whether the radiological reduction of scapholunate instability was maintained over time. The secondary objective was to evaluate whether functional improvement and patient-reported outcomes remained stable at long-term follow-up.

## 2 Methods

### 2.1 Selection criteria

This was a retrospective single-center case series of patients who underwent SLIC 2 ligamentoplasty between April 2014 and June 2019. In 2023, patients with a minimum follow-up of 5 years were contacted and invited for standardized long-term clinical and radiological reassessment. Eligible patients were those operated on for reducible chronic static scapholunate dissociation (stage 4 according to Garcia Elias^8^) using the SLIC 2 technique. Static scapholunate dissociation was defined as a scapholunate gap >3 mm on standard posteroanterior radiographs, associated with static carpal malalignment and a reducible deformity consistent with Garcia-Elias stage 4 instability. Surgical indication was based on persistent symptomatic chronic static scapholunate dissociation, characterized by pain during loading and/or functional limitation, and failure of at least 3 months of conservative treatment, including activity modification, splinting, analgesic or anti-inflammatory treatment, and/or hand therapy. As part of our local preoperative protocol, CT arthrography was systematically performed in addition to dynamic radiographic assessment. SLIC 2 ligamentoplasty was only indicated when CT arthrography confirmed the absence of significant chondral damage or advanced degenerative changes. Arthroscopic staging was not performed systematically and was not used as an inclusion criterion.

The exclusion criteria were the presence of associated wrist lesions and any stage other than stage 4 according to the Marc Garcia Elias classification.^8^ Exclusion criteria were poor adherence to the physiotherapy protocol (defined as <70% of supervised sessions, >3 missed consecutive appointments, other issues indicating inadequate participation or previous wrist surgery), non-compliance with postoperative follow-up or treatment, missing data in the medical records, and loss to follow-up.

Available short-term postoperative data at 1 year after surgery were collected from routine follow-up consultations. For the long-term analysis, patients were contacted in 2025 and invited for standardized clinical and radiological reassessment, provided that the minimum follow-up exceeded 5 years.

**Fig. 1:**
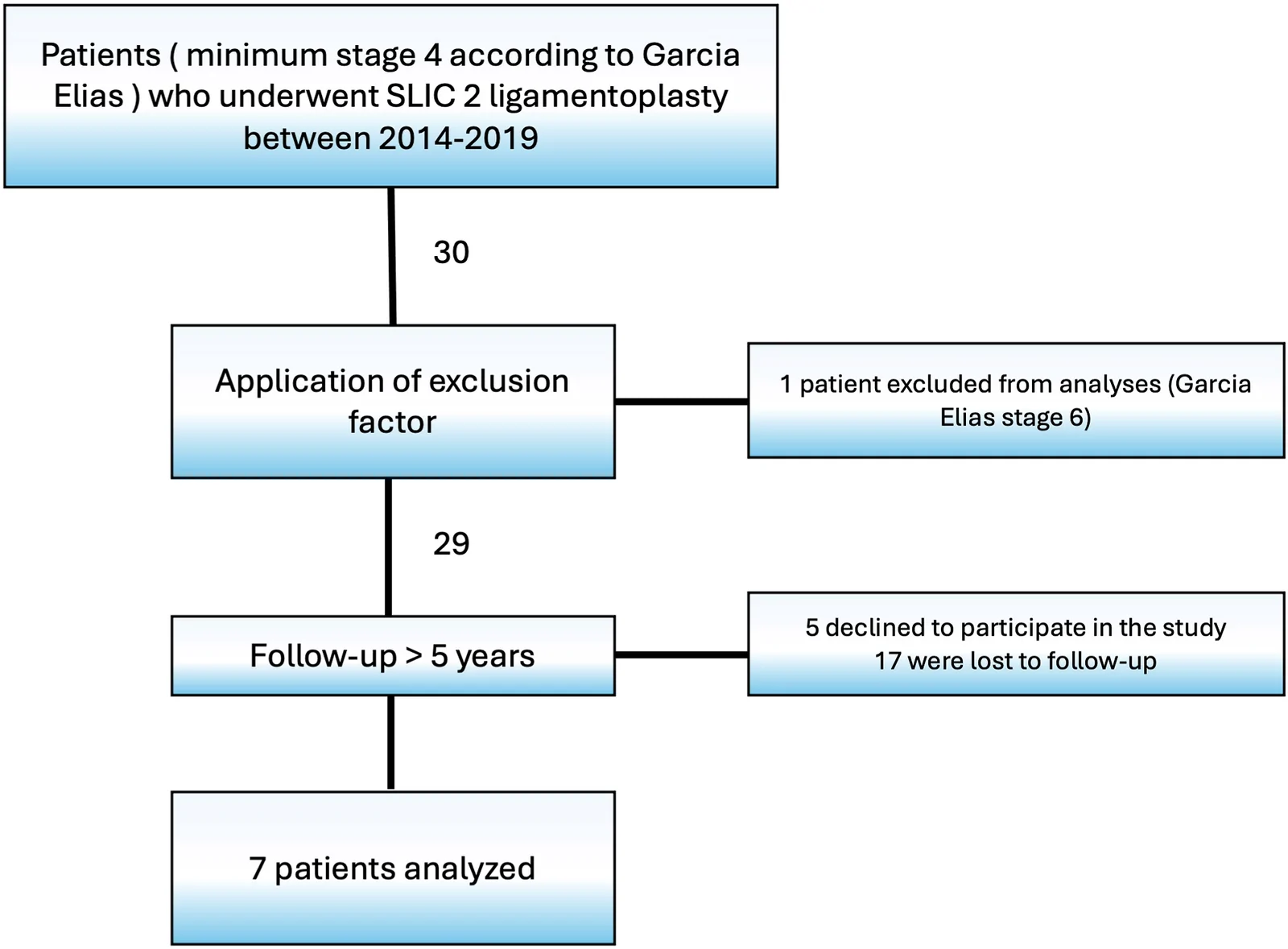
Flowchart.

Of the 56 potentially eligible patients screened, seven fulfilled all eligibility criteria and completed standardized clinical and radiological reassessment at a minimum follow-up of 5 years. The reasons for non-inclusion or exclusion are detailed in Fig. 1. All patients had chronic instability and underwent surgery at a mean of 9.3 months (range, 3–15 months) after injury or symptom onset. The mechanism was traumatic in 6 patients and atraumatic or uncertain in one patient. Generalized hyperlaxity was not systematically quantified using the Beighton score.

All patients were operated on from April 2014 to June 2019 in the same unit by two ‘‘Level 3 – Specialist – experienced’’ surgeons according to the Tang-Giddins classification,^16^^,^^17^ for SLIC ligamentoplasty 2.0. All patients provided written informed consent, and the study was approved by the local ethics committee (IRB: 2023-045795-3).

### 2.2 Radiological and clinical assessment

Radiological outcomes were assessed using standardized wrist radiographs obtained preoperatively, immediately postoperatively, at short-term follow-up, and at final follow-up. The evaluated parameters included the static scapholunate gap (mm) on posteroanterior views, the scapholunate angle (°) and capitolunate angle (°) on lateral views, and posterior scaphoid subluxation (mm). The posterior radioscaphoid angle (PRSA) (°) was also measured when available, as a marker of dorsal scaphoid translation and potential degenerative progression^18^ on available CT scans. The presence and progression of scapholunate advanced collapse (SLAC) arthritis were recorded at each radiographic assessment. Radiological classification and measurements were based on standardized unloaded posteroanterior and lateral wrist radiographs. The presence and progression of SLAC arthritis were assessed on serial posteroanterior and lateral radiographs according to the Watson and Ballet classification.^19^

Clinical and functional outcomes were evaluated preoperatively, at short-term follow-up, and at final follow-up. Pain was assessed using a visual analogue scale (VAS,/10) at rest and during effort. Objective clinical assessment included grip strength (Kg), compared with the contralateral side, and wrist range of motion, including flexion (°), extension (°), ulnar deviation (°), and radial deviation (°). Patient-reported and functional outcomes were assessed using the QuickDASH (/100), the Patient-Rated Wrist Evaluation score (PRWE,/100), and the Mayo Wrist Score (/100). These parameters were used to evaluate the long-term impact of the SLIC 2 procedure on pain, wrist function, strength, and motion. The clinical and radiological findings were documented in the medical records by the operating surgeon at each follow-up visit. Data extraction and collection for the present study were subsequently performed by an independent author who had not participated in the surgical procedures.

### 2.3 Statistical analysis

Qualitative variables were described as counts and percentages.

Quantitative variables were summarized as medians and interquartile ranges (IQRs). For radiological parameters, paired comparisons were performed between preoperative and final follow-up values, as well as between immediate postoperative and final follow-up values, using the Wilcoxon signed-rank test. The latter comparison was used to assess the maintenance of the initial surgical correction over time.

Given the small sample size, comparisons between preoperative and final follow-up values were performed using the Wilcoxon signed-rank test. All analyses were considered exploratory. The significance level was set at p < 0.05. Statistical analyses were performed using R software v4.0.0 (R Foundation for Statistical Computing, Vienna, Austria).

## 3 Surgical technique (Fig. 2)

The SLIC 2 procedure was performed through a dorsal approach centered on the radiocarpal joint. The extensor retinaculum was opened between the third and fourth compartments, followed by a Berger-type dorsal capsulotomy. A free palmaris longus tendon graft, or a hemi-flexor carpi radialis graft when the palmaris longus was absent, was harvested and pretensioned before implantation. A graft length of approximately 10 cm was used.

**Figure. 2:**
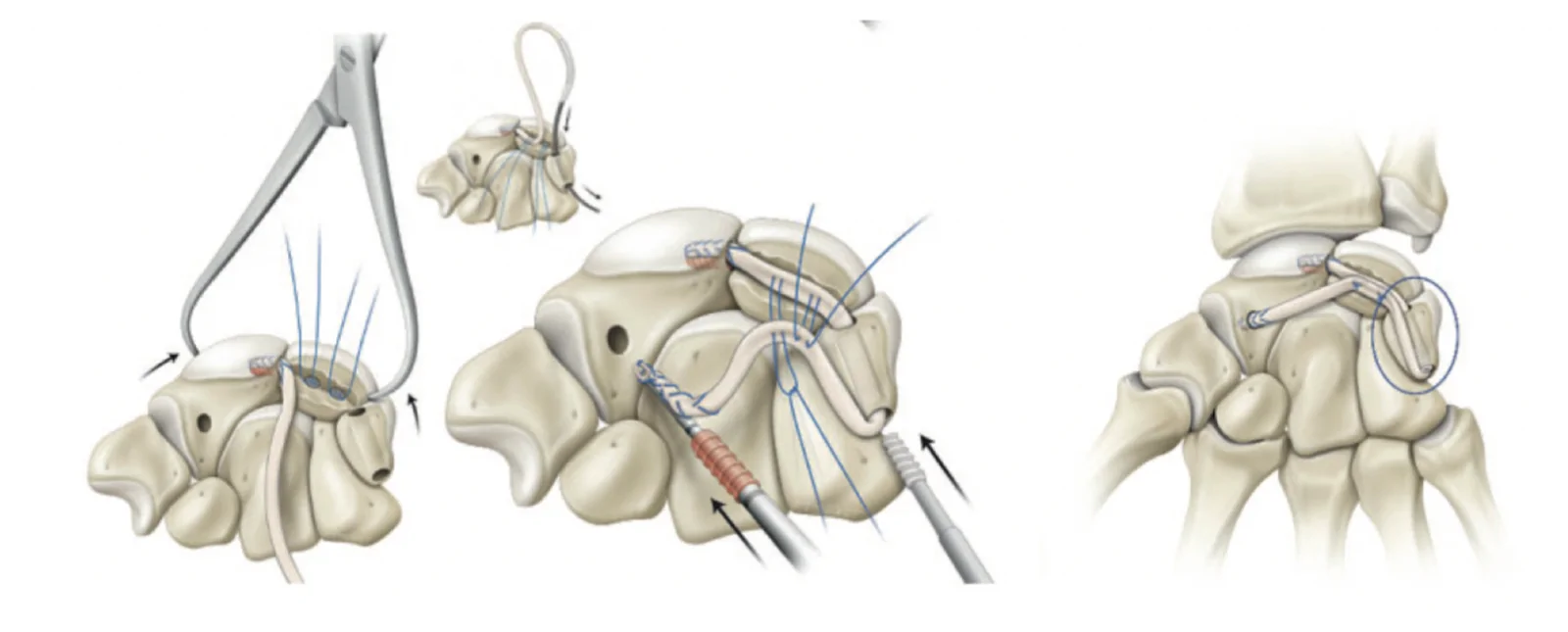
Scapholunate intercarpal ligamentoplasty 2.0 technique. *Schematic representation of the SLIC 2 procedure for chronic reducible static scapholunate dissociation. Through a dorsal approach, a free tendon graft is used to reconstruct two intercarpal stabilizing segments. First, the graft is anchored into a proximal blind scaphoid tunnel located at the former dorsal scapholunate ligament insertion, then fixed over a prepared dorsal lunate trench using suture anchors. After reduction of the scapholunate interval and temporary stabilization with scapholunate and scaphocapitate K-wires, the graft is passed through a transosseous triquetral tunnel and redirected toward a second distal scaphoid tunnel near the scaphotrapezotrapezoidal joint. This configuration creates a double ligamentoplasty designed to correct both the scapholunate gap and the DISI deformity by reinforcing the scapholunate, lunotriquetral, and scaphotriquetral relationships. Adapted from Athlani* et al.*, with permission.*

A first 10-mm-deep, 2.5-mm-diameter blind tunnel was created at the proximal pole of the scaphoid, corresponding to the former insertion of the dorsal scapholunate ligament. One end of the graft was secured within this tunnel using an interference fixation device. The dorsal aspect of the lunate was then prepared to create a shallow bony groove, and two suture anchors were positioned close to the scapholunate and lunotriquetral intervals, respectively.

A transosseous tunnel was created through the triquetrum, through which the graft was passed and redirected toward the second scaphoid tunnel. This triquetral passage allows the graft to reproduce the intercarpal stabilizing effect of the dorsal scaphotriquetral and lunotriquetral ligamentous complex while permitting appropriate graft tensioning. A 3.0-mm transosseous triquetral tunnel was drilled using a cannulated drill in the sagittal plane, with an approximately 20°–30° dorsopalmar obliquity from the lunotriquetral interval toward the ulnar border of the triquetrum. The scapholunate dissociation and DISI deformity were reduced under fluoroscopic control, and the correction was temporarily maintained using scapholunate and scaphocapitate K-wires. The free palmaris longus graft was pretensioned at 3 kg for 2 min on a dedicated tendon preparation workstation (Arthrex © Naples, Florida, USA) before implantation.

The graft was passed over the dorsal lunate and through the triquetral tunnel. Graft tension was adjusted by traction on its free end and secured at the triquetral tunnel while maintaining the carpal reduction. The graft was additionally secured within the dorsal lunate groove using the sutures from the anchors. The remaining graft was then redirected across the dorsal lunate toward a second blind tunnel created at the dorsal scaphoid isthmus, where final fixation was achieved using an interference fixation device. This configuration created the scapho-luno-triquetral and triquetro-luno-scaphoid limbs of the reconstruction.

After K-wire removal, patients underwent a supervised physiotherapy program focused on the progressive recovery of wrist range of motion until the sixth postoperative month. Strengthening and resisted activities were initiated only after six months.

## 4 Results

The final cohort included 6 men and 1 woman, with a mean age of 42.5 years (range, 30–58 years). The mean time to surgery was 9.3 months range (3 to 15). A total of 5 patients (71.4%) were manual workers or with a high range of motion of the wrist required for their occupation. Mean postoperative time off work was 4 months (range, 1–9 months). Four patients (57.1%) returned to their previous occupation, two required occupational reclassification, and one was declared unable to return to work. Mean follow-up was 74.8 months (range, 68–82 months). One patient presented with a preoperative static scapholunate gap of 2.3 mm, whereas all other patients had a gap >3 mm. Despite this lower gap value, this patient showed clinical and radiographic features consistent with reducible chronic static scapholunate instability and was therefore included in the cohort.

## 5 Radiological outcomes

Compared with immediate postoperative values, the median scapholunate gap increased from 2.0 mm (IQR, 1.8–2.1) to 4.2 mm (IQR, 4.0–4.4) (p = 0.016), the scapholunate angle increased from 50° (IQR, 49–64.5) to 80° (IQR, 76–86) (p = 0.016), the capitolunate angle worsened from −3° (IQR, −4.5–0) to −35° (IQR, −39 to −32.5) (p = 0.016), and posterior scaphoid subluxation increased from 0 mm (IQR, 0–0) to 1.0 mm (IQR, 0.3–2.8) (p = 0.031). When final follow-up values were compared with preoperative measurements, only the capitolunate angle showed a statistically significant difference, worsening from −22° (IQR, −29.5 to −12.5) to −35° (IQR, −39 to −32.5) (p = 0.016). One patient developed progressive SLAC arthritis during follow-up. Detailed radiological results are presented in Table 1.

**Table 1:** Comparison of radiographic results (median and interquartile range) for the study population (7 patients) between the preoperative, immediate postoperative, short-term follow-up, and last follow-up assessments.

Parameters	Preoperative	Immediate postoperative	Short-term follow-up	Last follow-up	P value^a^	P value^b^
Static SL gap (mm)	4.6 (3.2-6.0)	2.0 (1.8-2.1)	3.8 (3.4-4.0)	4.2 (4.0-4.4)	0.281	0.016
Scapholunate angle (°)	85 (78.5-87.5)	50 (49-64.5)	78 (68.5-79)	80 (76-86)	0.625	0.016
Capitolunate angle (°)	−22 (−29.5 to −12.5)	−3 (−4.5 to 0)	−14 (−18 to −4.5)	−35 (−39 to −32.5)	0.016	0.016
Posterior scaphoid subluxation (mm)	2.0 (1.9-3.3)	0 (0-0)	0 (0-0)	1.0 (0.3-2.8)	0.563	0.031
PRSA (°)	117 (110-123.5)	N/A	105.5 (102.5-107)	108 (106.5-108.5)	0.438	N/A
SLAC arthritis	0	0	1 SLAC 1	1 SLAC 3^c^		

SLAC: scapholunate advanced collapse; PRSA: posterior radioscaphoid angle.

a Evolution of the same patient who had SLAC 1 at short-term follow-up.

b Wilcoxon signed-rank test comparing preoperative and last follow-up values.

c Wilcoxon signed-rank test comparing immediate postoperative and last follow-up values.

## 6 Clinical and functional outcomes

At final follow-up, pain during effort decreased from a median of 5.5 (IQR, 3.8–7.2) preoperatively to 2 (IQR, 1–3) (p = 0.031). PRWE improved from 49.5 (IQR, 35.8–52.3) to 16 (IQR, 11.3–23.8) (p = 0.031), and Mayo Wrist Score improved from 70 (IQR, 67.5–77.5) to 80 (IQR, 72.5–82.5) (p = 0.016). Pain at rest and grip strength were preserved, while wrist range of motion showed a postoperative decrease, particularly in flexion, and QuickDASH improved without reaching statistical significance. Detailed clinical and functional outcomes are presented in Table 2.

**Table 2:** Comparison of clinical results (median and interquartile range) for the study population (7 patients) between the preoperative, short-term follow-up, and last follow-up assessments.

Parameters	Preoperative	Short-term follow-up	Last follow-up	P value^a^
Pain at rest (VAS) (/10)	2 (1-2.8)	0 (0-0)	1 (0-1)	0.250
Pain during effort (VAS) (/10)	5.5 (3.8-7.2)	3 (1-4)	2 (1-3)	0.031
Grip strength (kg)	30 (25-44)	38 (27-41)	34 (31-38.5)	1.000
Contralateral grip strength (kg)	42 (40-42.5)	40 (39-42)	43 (39.5-47)	0.750
Flexion (°)	70 (65-72.5)	40 (30-57.5)	50 (40-64)	0.109
Extension (°)	70 (70-70)	60 (50-67.5)	67 (64-68)	0.219
Ulnar deviation (°)	40 (27.5-40)	30 (20-35)	28 (25-32.5)	0.219
Radial deviation (°)	15 (15-17.5)	15 (10-20)	17 (10-18.5)	0.250
QDASH (/100)	43.18 (32.96-45.45)	18.18 (10.23-26.09)	18.20 (10.20-26.15)	0.219
PRWE (/100)	49.5 (35.8-52.3)	16.5 (11-17.5)	16 (11.3-23.8)	0.031
Mayo Wrist Score (/100)	70 (67.5-77.5)	80 (75-90)	80 (72.5-82.5)	0.016

VAS: visual analogue scale; QDASH: Quick Disabilities of the Arm, Shoulder and Hand; PRWE: Patient-Rated Wrist Evaluation.

a Wilcoxon signed-rank test comparing preoperative and last follow-up values.

## 7 Discussion

Chronic static scapholunate dissociation (SLD), particularly at stage 4 of the Garcia-Elias classification, remains a therapeutic challenge in the absence of chondral damage. In these advanced stages, ligamentoplasty aims to restore carpal alignment, relieve symptoms, and preserve wrist function, but the long-term reliability of many techniques remains controversial ^13^^,^^20^, ^21^, ^22^, ^23^, ^24^.

Our study investigates the outcomes of the SLIC 2 procedure in a homogeneous population of patients with chronic, static, reducible SLD at a minimum of five years follow-up.

In this series, no statistically significant differences were observed between preoperative measurements and those obtained at final follow-up except the capitolunate angle. This was particularly relevant for deformity patterns associated with degenerative progression, including posterior scaphoid subluxation and the PRSA. Overall, the initial radiological correction was progressively lost across the different parameters evaluated, suggesting a global recurrence of carpal malalignment over time. However, only the capitolunate angle showed a statistically significant worsening compared with the preoperative assessment. The discrepancy between radiological recurrence and preserved clinical function remains unexplained. As in other wrist conditions, substantial structural abnormalities may be clinically well tolerated, whereas limited radiological changes may be associated with significant symptoms.

Although the SLIC 2 technique achieved an initial correction of carpal malalignment, this correction was not maintained at long-term follow-up, suggesting that the reconstruction may not definitively restore normal scapholunate alignment over time. Only one patient developed progressive SLAC arthritis during follow-up, whereas the remaining patients showed no radiographic progression toward advanced degenerative changes. The rate of SLAC osteoarthritis varies from 5% for Chabas et al.^25^ to 23% for Garcia et al.^8^ The progression of SLAC in this single patient remains unexplained and may reflect the natural evolution of the underlying scapholunate pathology in the setting of recurrent carpal malalignment or a selection bias.

Despite this radiological recurrence, pain and functional outcomes remained improved at long-term follow-up. Pain during effort, Mayo Wrist Score, and PRWE showed significant improvement compared with the preoperative status, although these values remained relatively stable between the one-year assessment and the final follow-up.

Regarding wrist range of motion, no significant difference was observed between preoperative values and final follow-up. However, range of motion was reduced at one year postoperatively before progressively returning toward preoperative levels at long-term follow-up. This pattern, together with the partial loss of radiological correction, may suggest progressive relaxation of the reconstruction over time. Therefore, rather than providing definitive anatomical correction, the SLIC 2 technique may provide sustained clinical benefit during the prearthritic phase, despite partial recurrence of radiological deformity.

The original Scapholunate Intercarpal Ligamentoplasty (SLIC 1) was developed to treat chronic scapholunate dissociation using a tendon graft. However, it had limitations, particularly in achieving optimal graft tension and technical challenges at the triquetral step. To improve stability and simplify the procedure, a modified version—SLIC 2—was introduced. This version optimizes graft tension and fixation, better preserving intercarpal correction over time.^12^ Although tendon grafts may undergo progressive biological remodeling and ligamentization after reconstruction, wrist-specific evidence remains scarce, and the biological behavior of a free tendon graft after scapholunate reconstruction remains incompletely understood.^26^

Of particular interest, the only patient in our cohort who progressed to SLAC stage 3 had shown early radiographic signs of deterioration already at the short-term evaluation. This patient's PRSA worsened significantly from 117° preoperatively to 112° at short term, eventually reaching 120° at final follow-up—a value that exceeds the 114° threshold identified by Gondim Teixeira et al. as predictive of SLAC progression.^18^ This trajectory confirms the prognostic value of PRSA in anticipating degenerative changes even when conventional radiographic signs remain subtle in the early postoperative period.

Moreover, our population presented with a mean preoperative scapholunate angle of 82.7°, noticeably higher than in Athlani's group (approximately 75°), suggesting greater initial deformity. Despite this, our radiological correction—though partially lost over time—was comparable, with a final scapholunate angle of 81°. This recurrence is consistent with that observed in their series, reinforcing the idea that ligamentoplasty techniques may not always maintain anatomical correction in the long run, but can still offer durable clinical benefit.

In contrast, the 3LT technique, which reconstructs three ligaments using a more invasive approach, has shown more heterogeneous and less favorable results. In many series,^8^^,^^15^^,^^25^ 3LT patients reached nearly 71.2 points on the Mayo score and had a mean PRWE of 32.4, both notably lower than in our cohort. Complications such as postoperative stiffness, graft loosening, and even early SLAC changes were more frequently observed. Furthermore, functional deterioration was more likely when radiographic recurrence occurred, indicating less tolerance to residual deformity compared to the SLIC technique. Furthermore, this technique uses a slip of the flexor carpi radialis tendon. This involves sacrificing an active stabilizer of the carpus.^27^

The dorsal window approach described by Loisel et al. represents an attractive alternative to more extensive dorsal capsulotomies, as preservation of the dorsal intercarpal ligament may limit iatrogenic disruption of secondary carpal stabilizers.^28^ However, SLIC 2 ligamentoplasty requires direct exposure of the dorsal scaphoid, lunate, and triquetrum for tunnel preparation, graft passage, tensioning, and fixation. Consequently, although the window approach may be particularly suitable for procedures confined to the scapholunate interval, the broader exposure provided by the Berger approach remains useful for performing the complete SLIC 2 reconstruction.

The SLIC 2 procedure has specific characteristics that may distinguish it from other reconstructive techniques, although current evidence does not support the superiority of any single procedure. Compared with 3LT, SLIC 2 uses a free tendon graft and therefore generally preserves the flexor carpi radialis, an active stabilizer of the carpus, while providing a double intercarpal reconstruction involving the scaphoid, lunate, and triquetrum.^8^ In contrast, ANAFAB reconstruction addresses both the dorsal and volar components of scapholunate instability and therefore provides a more comprehensive anatomical reconstruction, but requires combined dorsal and volar approaches.^13^ Arthroscopic reconstruction techniques have the potential advantage of limiting soft-tissue disruption and preserving secondary stabilizers; however, they are technically demanding, and available evidence remains heterogeneous, particularly in chronic high-grade static instability.^29^ SLIC 2 therefore represents a dorsal reconstructive option that avoids a volar approach while allowing direct control of reduction, graft tension, and stabilization of the proximal carpal row. Nevertheless, it does not directly reconstruct the volar scapholunate component, which remains an important theoretical limitation.

Transosseous carpal tunnels are associated with specific concerns, including tunnel fracture, osteonecrosis, and carpal bone collapse, which may severely compromise the reconstruction and occasionally necessitate salvage procedures.^30^^,^^31^ However, the SLIC 2 technique limits transosseous bone tunneling compared with other reconstructive procedures. At the lunate, the graft is positioned within a shallow dorsal bony groove rather than through a complete transosseous tunnel, while the only complete transosseous tunnel is created through the triquetrum. No tunnel-related fracture, osteonecrosis, or bone collapse was observed in the present series. The preservation of the lunate and the absence of a complete transosseous lunate tunnel may theoretically reduce the risk of such complications. Furthermore, because the triquetral tunnel is located within the relatively preserved lunotriquetral component of the proximal carpal row, the mechanical consequences of this tunnel may differ from those of tunnels involving the scaphoid or lunate; however, this remains speculative and requires further investigation.

Several limitations should be acknowledged. First, this study was retrospective and conducted at a single center, which inherently limits the external validity and generalizability of the findings. The small sample size represents an additional major limitation, reducing statistical power and precluding robust subgroup or multivariable analyses. Although obtaining standardized reassessment after more than 5 years is challenging, particularly among patients with satisfactory clinical outcomes who may be less inclined to return for follow-up, the limited cohort size requires that the present findings be interpreted cautiously.

An additional limitation is the potential for selection and attrition bias related to the large proportion of patients who were unavailable for standardized long-term reassessment. Although the included cohort was homogeneous, patients with incomplete follow-up may have experienced different, and potentially less favorable, outcomes than those who returned for evaluation. Therefore, the clinical results observed in the present series may overestimate the overall outcomes of the initially treated population. This issue is particularly relevant in scapholunate reconstruction studies, as patients requiring revision or experiencing recurrent symptomatic instability may not be represented in conventional long-term follow-up cohorts. In a recent mid-term series of all-dorsal augmented scapholunate reconstruction, Goorens et al. reported that several patients were unavailable for final assessment because they had undergone revision surgery, emphasizing the importance of accounting for treatment failures when interpreting follow-up data.^32^

Third, a potential observer bias should be acknowledged. Clinical and radiological findings were documented during follow-up by the operating surgeons, and no blinded independent assessor was systematically involved in the longitudinal evaluation. Although data extraction for the present study was subsequently performed by an author who had not participated in the surgical procedures, this does not completely eliminate the potential for measurement or observer bias inherent to the original assessments.

Fourth, generalized ligamentous hyperlaxity could not be evaluated because the Beighton score was not systematically collected. Similarly, the absence of systematic stress radiographs or stress CT prevented assessment of the dynamic behavior of the scapholunate interval and dorsal scaphoid translation. This limitation remains relevant despite inclusion being restricted to static Garcia-Elias stage 4 dissociation.

The SLIC 2 technique does not directly address the volar component of scapholunate instability, unlike other reconstructive procedures that include a palmar stabilizing component.^13^^,^^33^ This partial reconstruction of the scapholunate complex may partially explain the progressive loss of radiological correction observed over time.

The absence of systematic stress radiographs or stress CT prevented evaluation of the dynamic behavior of the scapholunate interval and dorsal scaphoid translation. This limitation is relevant despite inclusion being restricted to static Garcia-Elias stage 4 dissociation.

Finally, although SLIC 2 remains a technically demanding procedure, the fixation material used in this technique did not appear to generate complications that would compromise subsequent salvage surgery. This point may be relevant when compared with other dorsal ligamentoplasty techniques, such as the all-dorsal reconstruction using SwiveLock anchors (Arthrex© Naples, Florida, USA), for which short-term bone lysis around the anchors has been reported in up to 50% of cases.^34^

In this homogeneous series of patients with chronic reducible static scapholunate dissociation, SLIC 2 ligamentoplasty provided sustained clinical improvement at a minimum follow-up of five years, despite partial loss of radiological correction over time. These findings suggest that SLIC 2 may remain a valuable option in selected stage 4 lesions, although larger comparative studies are needed to better define its long-term role and identify predictors of radiological or degenerative progression. Given the small and highly selected cohort, these findings should be interpreted cautiously and confirmed in larger studies, although they provide valuable insight into the long-term outcomes of SLIC 2 ligamentoplasty.

## Ethics

All patients provided written informed consent, and the study was approved by the local ethics committee (IRB: 2023-045795-3).

## AI declaration

Artificial intelligence was used solely to assist with language editing and manuscript preparation.

## Author statement

Rémy Dubian conceived the presented idea, supervised the findings of this work, wrote the manuscript.

Jean-Baptiste de Villeneuve Bargemon and Marc Leroy helped to write the manuscript and revised it.

Philippe Bellemère supervised the findings of this work, collected the data.

Ludovic Ardouin collected the data and supervised the presented idea.

## Declaration of competing interests

Last author Marc LEROY declare that he has conflict of interest with Arthrex®.

This research received no specific grant from any funding agency in the public, commercial, or not-for-profit sectors.
